# Characterization of novel hydrocarbon-degrading *Gordonia paraffinivorans* and *Gordonia sihwensis* strains isolated from composting

**DOI:** 10.1371/journal.pone.0215396

**Published:** 2019-04-18

**Authors:** Natalia Maria Silva, Aline Márcia Silva Araújo de Oliveira, Stefania Pegorin, Camila Escandura Giusti, Vitor Batista Ferrari, Deibs Barbosa, Layla Farage Martins, Carlos Morais, João Carlos Setubal, Suzan Pantaroto Vasconcellos, Aline Maria da Silva, Julio Cezar Franco de Oliveira, Renata Castiglioni Pascon, Cristina Viana-Niero

**Affiliations:** 1 Departamento de Microbiologia, Imunologia e Parasitologia, Universidade Federal de São Paulo, São Paulo, Brazil; 2 Departamento de Ciências Biológicas, Universidade Federal de São Paulo, Diadema, Brazil; 3 Departamento de Ciências Farmacêuticas da Universidade Federal de São Paulo, Diadema, Brazil; 4 Departamento de Bioquímica, Instituto de Química, Universidade de São Paulo, São Paulo, Brazil; Universita degli Studi di Milano-Bicocca, ITALY

## Abstract

Hydrocarbons are important environmental pollutants, and the isolation and characterization of new microorganisms with the ability to degrade these compounds are important for effective biodegradation. In this work we isolated and characterized several bacterial isolates from compost, a substrate rich in microbial diversity. The isolates were obtained from selective culture medium containing n-hexadecane, aiming to recover alkane-degraders. Six isolates identified as *Gordonia* by MALDI-TOF and 16S rRNA sequencing had the ability to degrade n-hexadecane in three days. Two isolates were selected for genomic and functional characterization, *Gordonia paraffinivorans* (MTZ052) and *Gordonia sihwensis* (MTZ096). The CG-MS results showed distinct n-hexadecane degradation rates for MTZ052 and MTZ096 (86% and 100% respectively). The genome sequence showed that MTZ052 encodes only one alkane degrading gene cluster, the CYP153 system, while MTZ096 harbors both the Alkane Hydroxylase (AH) and the CYP153 systems. qPCR showed that both gene clusters are induced by the presence of n-hexadecane in the growth medium, suggesting that *G*. *paraffinivorans* and *G*. *sihwensis* use these systems for degradation. Altogether, our results indicate that these *Gordonia* isolates have a good potential for biotransformation of hydrocarbons.

## Introduction

Crude oil and waste of petroleum derivatives are made of hydrocarbons and considered pollutants, which are difficult to treat and remove from the environment [1,2]. Aliphatic hydrocarbons (alkanes) are the major components of crude oil and can be subject to oxidative degradation by bacterial metabolism as a carbon source [3]. To circumvent the inertia of alkanes, some bacteria can activate these molecules through oxidation of their terminal methyl group, converting it into the corresponding primary alcohol. In the subsequent metabolic steps the methyl group is converted to an aldehyde and then to a fatty acid derivative, which enters the β-oxidation pathway to generate the central metabolite acetyl CoA [4,5]. The bacterial enzymatic systems that enable the activation and conversion of alkanes to harmless metabolic molecules comprise terminal monooxygenases such as the membrane-anchored AlkB family of alkane hydroxylases or soluble cytochrome CYP153A enzymes [6].

Among hydrocarbon-degrading actinobacteria [7,8], the genus *Gordonia* can uptake and consume aliphatic hydrocarbons [9,10]. Currently the *Gordonia* genus encompasses 39 valid species [11] but several of the isolates described as having the ability to degrade hydrocarbons were only identified at the genus level [12,13]. *Gordonia* spp. are widely distributed in nature and have caught attention due to their capabilities to degrade, transform, and synthesize organic compounds, thus having a promising biotechnological value [10,11,14].

Compost has been shown to carry rich microbial diversity and metabolic functions [15–18] and, as such, it has been explored as a source of biotechnologically relevant microorganisms [19–27]. The aim of this work was to isolate and characterize hydrocarbon-degrading bacteria from compost.

Here we report a genomic and functional characterization of two hydrocarbon-degrading strains isolated from composting after enrichment culture for actinobacteria using n-hexadecane as exogenous carbon source. The strains were unequivocally classified as *Gordonia paraffinivorans* and *Gordonia sihwensis*. Bioinformatics analyses showed that both strains have a CYP153 gene cluster; in addition, *G*. *sihwensis* also has the alkane hydroxylase (AH) system. Moreover, these gene clusters in the two species are induced by n-hexadecane. To the best of our knowledge, this is the first report showing that isolates of these *Gordonia* species have the ability to degrade n-hexadecane.

## Material and methods

### n-hexadecane enrichment culture from composting samples

Samples for the enrichment culture were obtained from a composting facility located at the São Paulo Zoo Park, São Paulo, Brazil [17]. To obtain a representative sample of the composting pile, sub-samples were collected from five different points (at the same depth in the four edges and center of the chamber) [28], pooled and thoroughly mixed. The samples were used for enrichment culture following two different protocols. In the first, named “direct culture”, 5 g of compost were suspended in 10 mL of sterile water and vigorously homogenized. After 1 h at room temperature, the mixture was centrifuged at 600 xg for 5 min, and 2.5 mL of supernatant was inoculated in selective medium as described below [27]. In the second protocol, which includes a decontamination step, 10 g of compost were suspended in 20 mL of sterile water, vigorously homogenized and then centrifuged at 600 xg for 5 min. The supernatant was transferred to a sterile tube, centrifuged at 8000 xg for 15 min and the pellet was subjected to a decontamination process with SDS/NaOH to enrich for resistant microorganisms such as actinobacteria [29]. The final precipitate was suspended in 2.5 mL of sterile water. The samples obtained by direct culture and the decontamination protocol were inoculated in 250 mL of M9 minimal medium containing 0.25% n-hexadecane (Sigma-Aldrich), 0.5% glucose and vitamin solution (200 mg p-aminobenzoic acid, 200 mg biotin, 200 mg folic acid, 200 mg nicotinic acid, 100 mg calcium D-pantothenate, 100 mg pyridoxine HCl, 100 mg riboflavin, 100 mg thiamine). Cultures were incubated at 30°C on a rotary shaker at 450 rpm during 2 days for direct culture and 7 days for the decontamination protocol-derived inoculum. Next, the cultures were centrifuged at 3220 xg for 10 min and the pellet was resuspended in 10 mL sterile water and subjected to centrifugation. This step was repeated once and the pellet was suspended on 250 mL of M9 minimal medium containing 0.5% of n-hexadecane without glucose and without vitamin solution followed by incubation at 30°C on a rotary shaker at 450 rpm during 2 days for the direct culture and 7 days for the decontamination protocol-derived inoculum. The bacterial pellet was recovered and washed as described above before starting the third cultivation step in M9 minimal medium containing 1% n-hexadecane in the same conditions. The final cultures were centrifuged and the pellets were inoculated on M9 medium agar plates that had been covered with a homogeneous layer of 100 μL 1% n-hexadecane, and incubated at 30°C until colonies were visible. Each one of the bacterial colonies were transferred to new agar plates and after growth they were stored at -80°C in glycerol suspensions.

### Microbial identification by MALDI-TOF MS and 16S rRNA gene sequencing

Protein extraction from each microbial isolate for MALDI-TOF MS (Matrix-Assisted Laser Desorption/Ionization Time-of-Flight Mass Spectrometry) analysis was performed by the ethanol/formic acid methodology as recommended by Bruker Daltonics. Briefly, after extraction, 1 μL of each sample was placed in triplicate on a 384-well desorption plate, overlaid with 1 μL of saturated solution of a α-cyano-4-hydroxycinnamic acid matrix (Sigma, USA) and dried at room temperature. The automatic acquisition of the spectra was performed using a Microflex LT mass spectrometer at 337 nm with the FlexControl software (Bruker Daltonics) in a linear positive ion mode at a laser frequency of 40 Hz and mass range of 2000 to 20,000 Da. Bacterial Test Standard (*E*. *coli* protein extract; Bruker Daltonics) was used for calibration. The analysis of the generated spectra was performed by BioTyper software version 3.0 (Bruker Daltonics), which compares the obtained spectra with the available profile in the database, generating a score value from 0 to 3. Scores were interpreted according to Bruker Daltonics as follows: < 1.700: not identified; 1.7 to 1.999: probable genus identification; 2.0 to 2.299: probable species identification; and ≥ 2.300: reliable species identification.

For 16S rRNA gene sequencing, bacterial genomic DNA was purified using the QIAamp DNA Mini Kit (Qiagen) and used as template for amplification of a 862 bp fragment with universal primers 530F (GTGCCAGCMGCCGCGG) and 1392R (ACGGGCGGTGTGTRC) as previously described [30]. Sequencing was performed in an ABI Prism 3100 Genetic Analyzer (Applied Biosystems) using the same primers used for amplification. The 16S rRNA gene sequences were compared to sequences deposited in GenBank using BLAST [31] and were deposited in GenBank (http://www.ncbi.nlm.nih.gov/genbank) under accession numbers MH611355 –MH611360.

### Qualitative assay of n-hexadecane degradation with 2,6-DCPIP

The 2,6-DCPIP (2,6-dichlorophenol indophenol) assay was performed on Bushnell-Hass (BH) mineral medium previously described [32,33] with minor modifications, as follows. The isolates were pre-cultured in Luria Bertani (LB) medium at 30°C for 48 hours and then centrifuged at 4000 xg for 5 min and washed with 0.9% sterile saline. The final bacterial suspension was adjusted to 1.0 (OD_660nm_), and 100 μL was transferred to a 1.5 mL tube containing 836.5 μL of BH medium, 53.5 μL of 2,6-DCPIP solution (375 μg/mL), and 10 μL n-hexadecane (Sigma) to final concentration of 1% (v/v). The assay was incubated at 30°C on a rotary shaker at 150 rpm for 21 days and observed every 24 hours. As negative controls for this assay, incubations were performed only with: 1) BH medium and DCPIP; 2) BH medium, DCPIP and n-hexadecane; 3) culture medium, DCPIP and bacterial suspension; and 4) BH medium, DCPIP and bacterial suspension inactivated by autoclaving. The assay was considered positive for n-hexadecane degradation if color changed from blue to colorless. No change in color was considered as negative result. *Mycobacterium vanbaalenii* PYR1 DSMZ7251 and *Escherichia coli* ATCC8739 strains were used as positive and negative controls, respectively.

### Evaluation of n-hexadecane degradation by GC-MS

Quantitative n-hexadecane biodegradation was analyzed using GC-MS (Gas chromatography-mass spectrometry analysis). *Gordonia* isolates were inoculated (10%, v/v) in BH mineral medium (pH 7 ± 0.2) containing n-hexadecane (1%, v/v) at 28°C in a rotary shaker at 150 rpm for 28 days. The assays were monitored at times 0, 72 h, 7 days, 14 days, 21 and 28 days as previously described [34], with modifications. Negative control corresponds to the mineral medium BH containing n-hexadecane without microorganisms. At the time of collection, 10 mL samples of the bacterial culture were transferred to an Agilent Chem Elut Cartridge System, and the same volume of ethyl acetate (Synth, 98%) was added. The filtrate (hexadecane and other organic compounds eluted with ethyl acetate) was collected and analyzed by GC–MS (Agilent 5975C Series GC/MSD) with the following conditions: injector at split (10:1) mode; He as carrier gas, at a 1 mL/ min flow; DB-5 MS column (30 m x 0.25 mm x 0.25 mm; 5% phenyl methyl silicone as stationary phase). The following program was used: initial temperature (100°C), heating rate (20°C/min), final temperature (290°C), injector temperature (240°C), detector temperature (280°C), temperature in the transfer line or interface (280°C), mass range from (50 to 700 Da). All the data were acquired using the SCAN mode. Naphthalene solution (3.9 mM) (Sigma-Aldrich, 98%) was used as internal standard. The biodegradation index for each bacterial isolate (according n-hexadecane degradation) was calculated as described previously [35].

### Genome sequencing, assembly and annotation

The genomes of strains MTZ052 and MTZ096 were sequenced using the Illumina/MiSeq Plataform. Shotgun genomic libraries were prepared using Illumina Nextera DNA library preparation kit with total DNA input of ~35 ng. The resulting DNA fragment libraries were cleaned up with Agencourt AMPure XP beads (Beckman Coulter) and fragment size within the range of 400–700 bp was verified by running the 2100 Bioanalyzer (Agilent) using the Agilent High Sensitivity DNA chip. Fragment library quantification was performed with KAPA Library Quantification Kit and sequencing run was done with the MiSeq Reagent kit v2 (500-cycle format, paired-end (PE) reads). On average, Illumina PE read1 and read2 presented, respectively, ≥ 80% and ≥ 75% of bases with quality score at least 30 (Q30). Raw reads were assembled with MIRA 4 [36] and ABACAS [37]. Completeness of the genome was verified with CheckM [38]. Annotation was carried out by the NCBI Prokaryotic Genome Annotation Pipeline (https://www.ncbi.nlm.nih.gov/genome/annotation_prok/). The genomes have been deposited in GenBank and are available under the following identifiers: Bioproject PRJNA287716; Biosamples SAMN03785435 (MTZ052) and SAMN03785436 (MTZ096); accessions LHVR00000000 and LHVQ00000000. The genomes were also submitted to the IMG/M annotation pipeline [39] and are available under the following IMG identifiers: 2695421033 (MTZ052) and 2695421034 (MTZ096).

### Bioinformatics analyses

The Digital DNA-DNA hybridization (dDDH) values for the *Gordonia* isolates were calculated using the Genome-to-Genome Distance Calculator (GGDC) 2.1 web service [40]. The Mummer-implemented Average Nucleotide Identity (ANIm) [41,42] was calculated using a public available script (https://github.com/widdowquinn/pyani).

We used the program Get_homologues [43] to infer gene families in *Gordonia* genomes and in two outgroup genomes publicly available. Phylogenetic reconstruction was done by a supermatrix approach with IQ-tree [44] and visualized with ggtree [45], using 57 single-copy gene families identified with Get_homologues. The two outgroup species were identified as the closest to the *Gordonia* genus by preliminary BLAST searches.

The amino acid sequences encoded by *Alk*, *RubA3*, *RubA4*, *RubB*, *AlkU* (AH) and *Fer*, *Cyp153*, *Fer Red* (CYP153) of *Gordonia sihwensis* NBRC 108236 and *Gordonia paraffinivorans* NBRC 108238 were used to query the MTZ052 and MTZ096 genomes with BLASTp and tBLASTn [31]. The coding sequences of *G*. *paraffinivorans* MTZ052 and *G*. *sihwensis* MTZ096 alkane gene clusters AH and CYP153 were used to query the genomes of various groups of bacteria.

### RNA extraction and cDNA synthesis

An isolated bacterial colony was inoculated in 30 mL of Luria Bertani (LB) in a 250 mL Erlenmeyer flask and incubated at 30°C with 150 rpm rotation overnight. The inoculum was transferred to a 50 mL plastic tube and centrifuged at 3220 xg for 15 minutes. The supernatant was discarded and the cells pellet was washed twice with 50 mL of milli-Q sterile water. The pellet was resuspended in 5 mL of sterile milli-Q water and the cell number was adjusted to 5 x 10^9^ according to McFarland standard. The cells were transferred to 10 mL BH medium containing the carbon sources: 1% n-hexadecane (inducing condition) or 2% fructose or dextrose (reference condition). The cultures were incubated in 50 mL Erlenmeyer flasks at 30°C and 150 rpm rotation for 2 hours. Three biological replicates were made for each treatment. RNA extraction was performed with the "Midi RNeasy Kit (Qiagen) according to the manufacturer's recommendations, with an addition of 60 minutes of incubation in a water bath with 50 mg/mL lysozyme at 37°C for cell lysis improvement. A digestion was made using “DNase I, RNase free” (Roche) to eliminate residual genomic DNA after the RNA extraction, according to the manufacturer's instructions. A PCR amplification of 16S rRNA using as template RNA and gDNA (positive control) was conducted to evaluate the efficiency of the DNase treatment. The amplification products were separated by electrophoresis in 0.8% agarose gel, stained with 0.25 μg/mL of ethidium bromide and visualized in ultraviolet light with orange filter. cDNA synthesis was made with the RevertAid kit First Strand cDNA (Thermo Scientific) using hexamers as initiators and the Reverse Transcriptase enzyme.

### qPCR

Quantification was done by quantitative PCR in real-time PCR System Thermal Cycler StepOnePlus (Applied Biosystems) in Fast Optical MicroAmp plates 96-Well Reaction Plate with Barcode, using SyberGreen as intercalating agent, according to the manufacturer's protocol. Due to the high content of CG (cytosine/guanine) present in the samples, 5% dimethyl sulfoxide (DMSO) was added to the reaction.

The cDNAs were diluted 1:10 in ultra-pure sterile water. The gene-specific primers used are described in S1 Table. As endogenous control we have used the 16S rRNA gene, which is often used in bacterial gene expression studies [9,46–49]. The amplifications conditions were adjusted through StepOne V2.3 Software using the default settings for the Cycle Threshold (CT), and assays were performed in triplicate for each sample. Gene induction in the presence of n-hexadecane (inducing condition) relative to the reference condition (fructose or dextrose) was calculated by the 2^-ΔΔCT^ method [50]. Statistically significant differences were calculated by ANOVA considering *p* values < 0.05 using Prisma Software.

## Results

### Bacterial isolation and identification

The 44 bacterial isolates from composting samples recovered after enrichment culture with n-hexadecane are listed in Table 1. Out of these, 25 were isolated using the direct culture protocol and 19 isolates were obtained using a protocol that includes a decontamination step [29] to favor actinobacteria selection (see Methods). While 36 isolates could be identified by MALDI-TOF MS at the genus (*Acinetobacter*, *Pseudomonas*, *Stenotrophomonas* and *Gordonia*) or species level (*Klebsiella pneumoniae* and *Bacillus shackletonii)*, six isolates were not identified because they presented a score lower than 1.7 (Table 1). Therefore, these six isolates were submitted to 16S rRNA gene sequencing and were identified as *Aquamicrobium* spp. (MTZ026 and MTZ027), *Chryseobacterium* spp. (MTZ062), *Gordonia paraffinivorans* (MTZ052) and *Gordonia cholesterolivorans* or *sihwensis* (MTZ095 and MTZ096) (Table 1). MTZ095 and MTZ096 were further identified as *G*. *sihwensis* based on their ability to utilize both citrate and D-galactose as carbon sources. *G*. *sihwensis* is known to use both carbon sources, unlike *G*. *cholesterolivorans* [51].

**Table 1:** Identification of the isolates by MALDI TOF-MS or 16S rRNA gene sequencing.

Isolates	Cultivation protocol	MALDI-TOF MS (score)	16S rRNA gene sequencing GenBank Accession best hit (% identity)
MTZ025	DC	*Acinetobacter* sp. (2.008)	-
MTZ028	DC	*Acinetobacter* sp. (2.041)	-
MTZ029	DC	*Acinetobacter* sp. (2.007)	-
MTZ030	DC	*Acinetobacter* sp. (2.000)	-
MTZ031	DC	*Acinetobacter* sp. (2.006)	-
MTZ032	DC	*Acinetobacter* sp. (2.017)	-
MTZ033	DC	*Acinetobacter* sp. (2.126)	-
MTZ035	DC	*Acinetobacter* sp. (2.034)	-
MTZ037	DC	*Acinetobacter* sp. (2.074)	-
MTZ038	DC	*Acinetobacter* sp. (2.004)	-
MTZ039	DC	*Acinetobacter* sp. (2.011)	-
MTZ054	D	*Acinetobacter* sp. (2.045)	-
MTZ058	DC	*Acinetobacter* sp. (2.078)	-
MTZ059	DC	*Acinetobacter* sp. (2.112)	-
MTZ060	DC	*Acinetobacter* sp. (2.009)	-
MTZ061	DC	*Acinetobacter* sp. (2.295)	-
MTZ063	DC	*Acinetobacter* sp. (2.172)	-
MTZ081	D	*Acinetobacter* sp. (2.196)	-
MTZ082	DC	*Acinetobacter* sp. (2.166)	-
MTZ084	DC	*Acinetobacter* sp. (2.075)	-
MTZ094	D	*Bacillus shackletoni* (2.319)	-
MTZ080	D	*Elizabethkingia* sp. (2.191)	-
MTZ053	D	*Gordonia* sp. (1.823) ^**§**^	-
MTZ055	D	*Gordonia* sp. (2.022) ^**§**^	-
MTZ056	D	*Gordonia* sp. (2.078) ^**§**^	-
MTZ036	DC	*Klebsiella pneumoniae* (2.361)	-
MTZ079	D	*Pandorea* sp. (2.133)	-
MTZ034	DC	*Pseudomonas* sp. (2.012)	-
MTZ078	D	*Pseudomonas* sp. (2.087)	-
MTZ083	DC	*Pseudomonas* sp. (2.070)	-
MTZ089	D	*Pseudomonas* sp. (2.031)	-
MTZ090	D	*Pseudomonas* sp. (2.124)	-
MTZ106	DC	*Pseudomonas* sp. (2.008)	-
MTZ048	D	*Stenotrophomonas* sp. (2.138)	-
MTZ049	D	*Stenotrophomonas* sp. (2.139)	-
MTZ050	D	*Stenotrophomonas* sp. (2.140)	-
MTZ051	D	*Stenotrophomonas* sp. (2.141)	-
MTZ057	D	*Stenotrophomonas* sp. (2.026)	-
MTZ026	DC	NI (<1.7)	*Aquamicrobium lusatiense/defluvii* KM210272.1/ KU163265.1 (100/99)
MTZ027	DC	NI (<1.7)	*Aquamicrobium lusatiense/defluvii* KM210272.1/ KU163265.1 (100/99)
MTZ052	D	NI (<1.7)	*Gordonia paraffinivorans* ^**§**^ JN627166.1 (100)
MTZ062	DC	NI (<1.7)	*Chryseobacterium* sp KX350224.1/ KP893287.1/ LC040953.1/ EU169201.1/ KP875413.1 (99)
MTZ095	D	NI (<1.7)	*Gordonia sihwensis/cholesterolivorans* ^**§**^ EU862318.1/ NR044445.1 (100/99)
MTZ096	D	NI (<1.7)	*Gordonia sihwensis/cholesterolivorans* ^***§***^ EU862318.1/ NR044445.1 (100/99)

DC: direct culture; D: decontamination protocol. Isolates tested positive for the ability to biodegrade the n-hexadecane are indicated by §.

### Qualitative evaluation of n-hexadecane degradation by selected isolates

Further characterization of the isolates regarding their ability to degrade hydrocarbons was performed on 9 out of 44 isolates that were identified: *Aquamicrobium spp*. (MTZ026 and MTZ027), *B*. *shackletonii* (MTZ094), *Gordonia spp*. *(*MTZ053, MTZ055 and MTZ056), *G*. *paraffinivorans* (MTZ052) and *G*. *sihwensis* (MTZ095 and MTZ096). These nine isolates were analyzed by the qualitative n-hexadecane degradation assay with 2,6-DCPIP. The results showed that all six isolates of *Gordonia* have the ability to biodegrade the n-hexadecane in three days (Table 1). Negative controls remained unchanged up to 21 days of incubation, confirming that no false positive reaction occurred among the assay components during this period. The isolates *Aquamicrobium* (MTZ026 and MTZ027) and *Bacillus shackletonii* (MTZ094) presented negative results for this test.

### Genome sequencing and in silico identification of genetic determinants involved in n-alkane-degradation

Guided by the qualitative evaluation of n-hexadecane degradation, two isolates (MTZ052 and MTZ096) were selected for genome sequencing using Illumina/MiSeq platform. Assembly of the reads resulted in draft genomes with sizes of ~4.9 Mb and ~3.9 Mb, respectively, for MTZ052 and MTZ096 (Table 2). The genome size of a *G*. *paraffinivorans* NBRC 108238 (accession number BAOQ01000008) has been reported at 4.63 Mb while the two genome sequences of *G*. *sihwensis* strains NBRC 108236 (accession number NZ_BANU00000000) and S9 (accession number JZDP01000000) have approximately 4.1 Mb each. *In silico* DNA-DNA hybridization (dDDH) and Average Nucleotide Identity (ANIm) values of the isolates MTZ052 and MTZ096 in comparison to *G*. *paraffinivorans* and *G*. *sihwensis* public genomes (S2 Table) confirm the assignment of MTZ052 and MTZ096 as *G*. *paraffinivorans* and *G*. *sihwensis*, respectively, according to the current criteria for taxonomic assignment based on whole genome sequencing [52,53].

**Table 2:** Sequencing, assembly and annotation results for MTZ052 and MTZ096 genomes.

General features	*G*. *paraffinivorans* MTZ052	*G*. *sihwensis* MTZ096
Number of paired-end reads (Illumina/MiSeq)	2,739,798	743,577
Sequencing coverage	168.5	51.2
Number of contigs	300	179
N50	164,877	173,966
Genome size (bp)	4,889,675	3,979,216
Completeness (%)	99.46	99.76
G+C (%)	68.77	68.27
Number of protein-coding genes	4,389	3,655
Number of rRNA operons*	1	1
Number of tRNAs	49	54
Number of other RNA genes	10	17

* Number of rRNA operons determined by manual curation of NCBI Prokaryotic Genome Annotation results.

**Fig 1:**
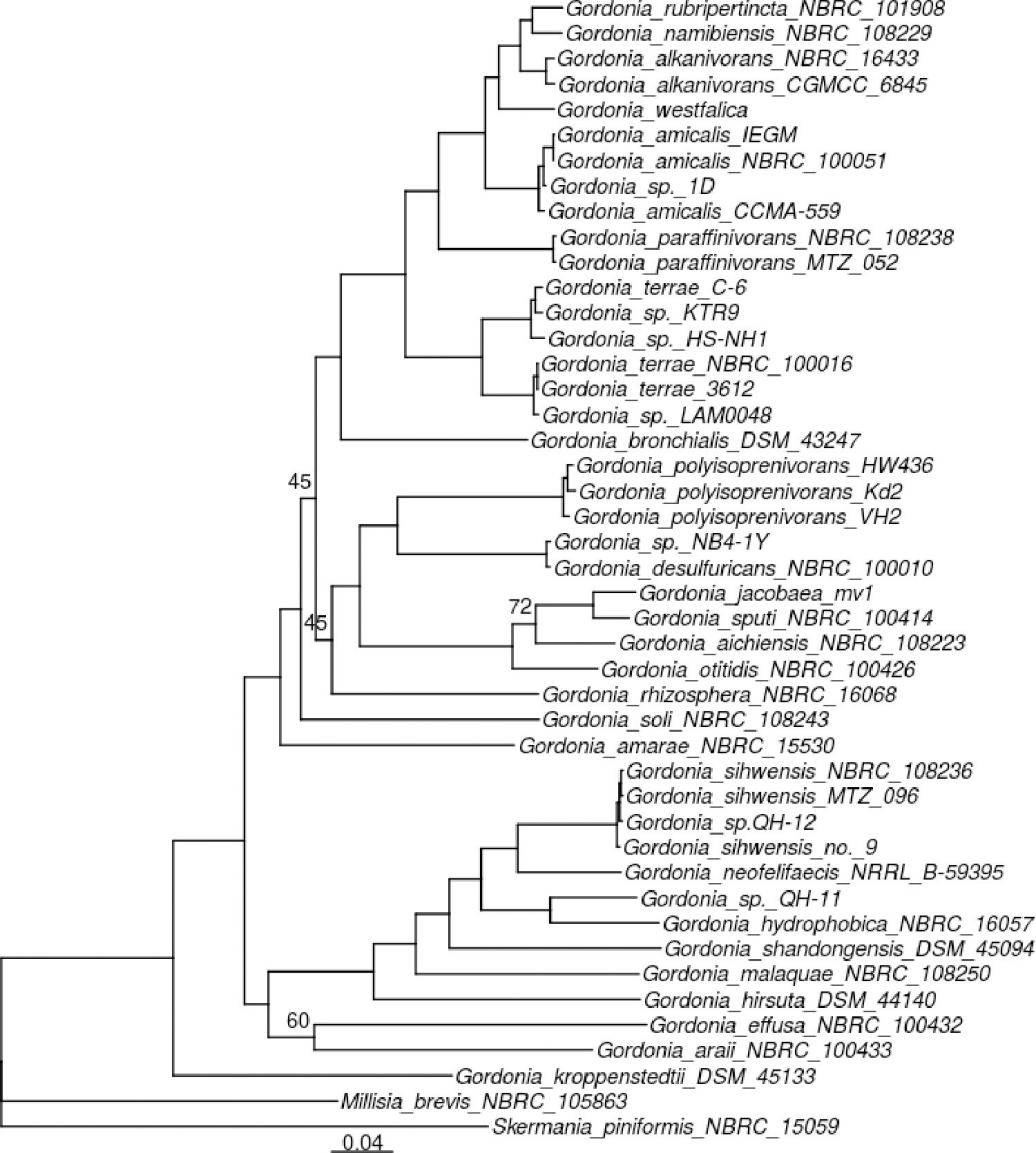
Phylogenetic analysis of MTZ052 and MTZ096. Maximum likelihood-based inference phylogenetic trees were reconstructed based on 57 gene families shared by the indicated bacterial species. *Millisia brevis* NBRC 105863 and *Skermania piniformis* NBRC 15059 were used as an outgroup. Branch bootstrap values are at least 80%, unless otherwise indicated.

Further confirmation of MTZ052 and MTZ096, respectively, as *G*. *paraffinivorans* and *G*. *sihwensis* was obtained by phylogenetic analysis using using 57 homologous genes of *Gordonia* species (S3 Table). As shown in the phylogenetic tree (Fig 1) MTZ052 clustered with the type strain *G*. *paraffinivorans* NBRC 108238 and MTZ096 clustered with *G*. *sihwensis* NBRC 108236.

**Fig 2:**
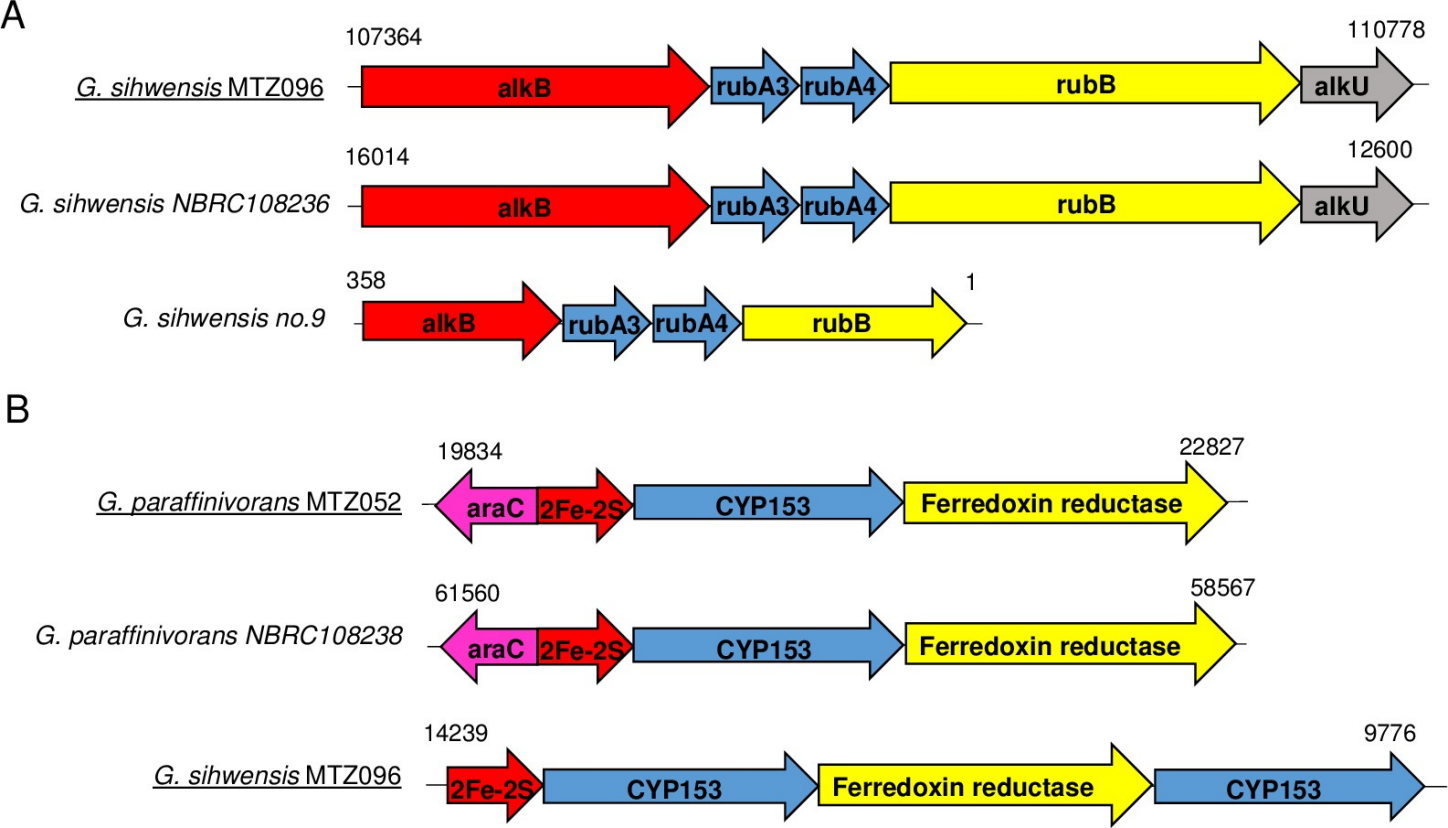
Schematic comparison of the two main gene clusters for alkane degradation in MTZ052 and MTZ096. (A) Alkane hydroxylase cluster genes: alkane monooxygenase (red), rubredoxin (blue) and reductase (yellow). (B) CYP153 cluster genes: 2Fe-2S ferredoxin (red), cytochrome P450 (blue) and ferredoxin reductase (yellow). Transcriptional regulators colored in gray and pink are indicated. Comparison between the strains studied in this work (underlined) and available strains in IMG/M are shown.

Next, the MTZ052 and MTZ096 genomes were screened for genes related to n-alkane degradation. One of the main gene clusters related to this phenotype is the alkane hydroxylase (AH) [9]. This cluster usually contains five genes (*alkB*, *rubA3*, *rubA4*, *rub* and *alkU*) as depicted in Fig 2. We found out that MTZ052 does not encode this cluster, whereas the MTZ096 genome encodes these five proteins with amino acid sequence similarities to NBRC 108236 (*G*. *sihwensis*), as shown in Table 3.

**Table 3:** Similarities among amino acid sequences encoded by the AH system in MTZ096 and NBRC 108236 (*G*. *sihwensis*).

Amino acid sequence (AH system)	% similarity
	MTZ096/NBRC108236
AlkB	100
RubA3	100
RubA4	100
RubB (rubredoxyn reductase)	99.5
AlkU	99.5

The CYP153 gene cluster (*fer*, *cyp153* and *fer red*) has also been described as important for n-alkane degradation, therefore we used the amino acid sequences from *Gordonia paraffinivorans* NBRC 108238 to search both MTZ isolate genomes. As shown in Fig 2, both strains showed high sequence similarities to the proteins encoded by this cluster (Table 4). We concluded that the MTZ052 (*G*. *paraffinivorans*) genome has only one of the clusters related to n-alkane degradation, while MTZ096 (*G*. *sihwensis*) genome encodes both clusters.

**Table 4:** Similarities among amino acid sequences encoded by the CYP153 gene clusters in MTZ052, MTZ096 and NBRC 108238 (*G*. *paraffinivorans*).

Amino acid sequence (CYP153 system)	% similarity	
	MTZ052/MTZ096	MTZ052/NBRC108238
Fer	75	100
CYP153	83	99.4
Fer Red	73	99.5

### Quantitative assay for MTZ052 and MTZ096 ability of hydrocarbon degradation

As shown above the two isolates *G*. *paraffinivorans* (MTZ052) and *G*. *sihwensis* (MTZ096) were able to grow in the presence of n-hexadecane, and their respective genomes encode AH and CYP153 gene clusters related to hydrocarbon degradation. Additional confirmation that these isolates can consume n-hexadecane over a period of time was obtained by quantitative GC/MS analyses (S1 Fig). Upon 28 days of cultivation in n-hexadecane as sole carbon source, it was possible to observe hexadecane biodegradation indexes of 86% and 100%, for MTZ052 and MTZ096, respectively. It is important to highlight that for both isolates, biodegradation indexes higher than 50% were observed already after 72h of microbial culture (Table 5). The negative control did not show degradation during the time course (S1 Fig).

**Table 5:** Biodegradation indexes of n-hexadecane of MTZ052 and MTZ096 strains during 28 days of chromatographic monitoring.

Monitoring Times	*G*. *paraffinivorans* MTZ052	*G*. *sihwensis* MTZ096
72 h	51.7%	71.6%
7 days	64.1%	86.4%
14 days	82.4%	99.9%
28 days	86.3%	100%

### Expression of CYP153 and AH gene clusters is induced by n-hexadecane

**Fig 3:**
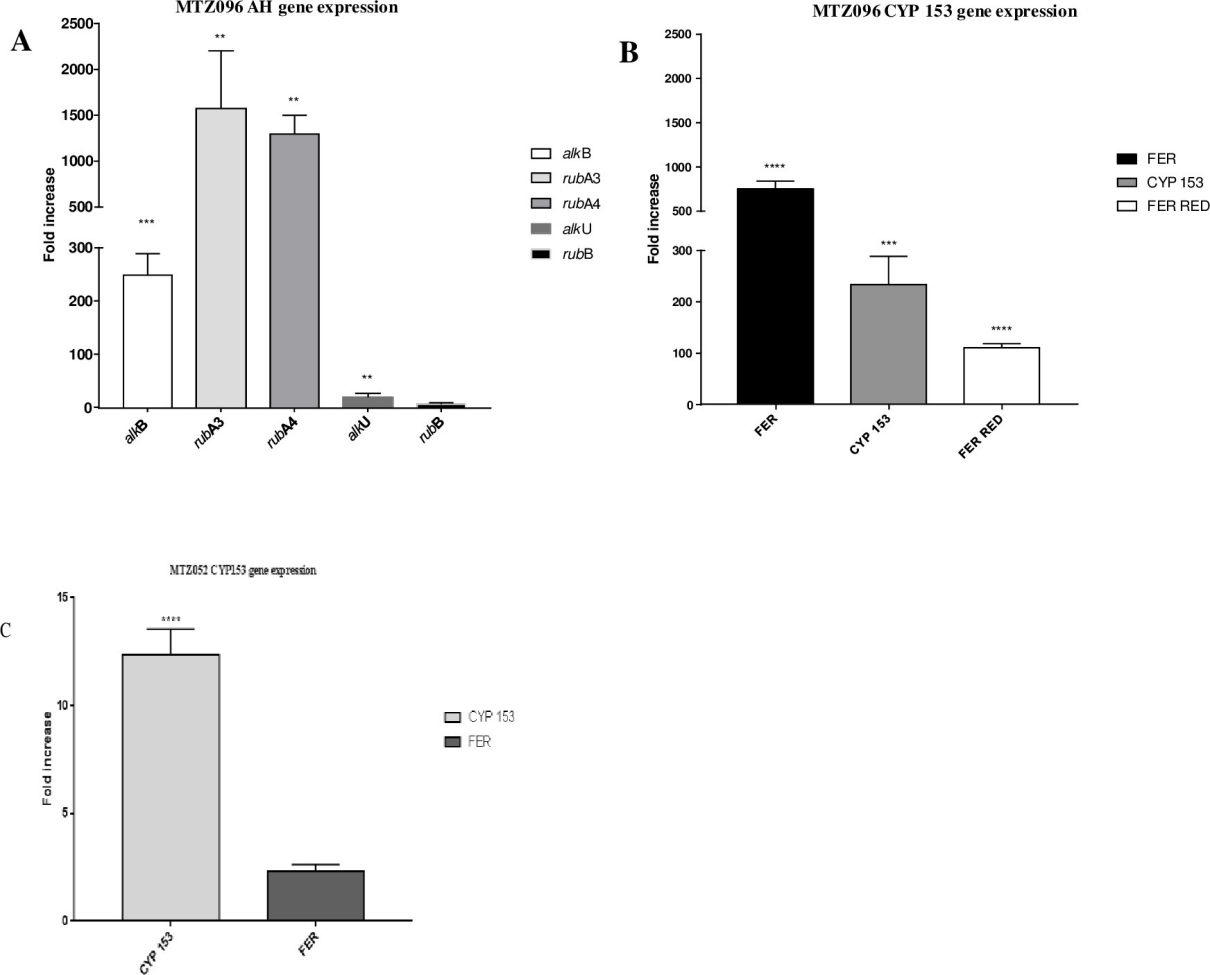
Transcriptional pattern by real-time PCR. AH, (B) CYP153 gene clusters in *G*. *sihwensis* MTZ096 and (C) transcriptional pattern of CYP153 gene cluster in *G*. *paraffinivorans* MTZ052.

Since *G*. *paraffinivorans* and *G*. *sihwensis* are capable of n-hexadecane degradation over a short period of time, we evaluated whether the CYP153 and AH gene clusters that were found in the genomes of each strain would be involved in this process. Thus, the transcription pattern for each of the genes within these clusters was evaluated by RT-qPCR. As shown in Fig 3A and 3B, for MTZ096, except for *rubB*, all genes in both clusters were highly induced in the presence of n-hexadecane after two hours of induction (248, 1567, 1296 and 2-fold induction, respectively, for *alkB*, *rubA3*, *rubA4* and *alkU*; and 750, 232 and 110 fold induction for Fer, CYP153 and Fer Red, respectively; p < 0.05). For MTZ052, which encodes only the CYP153 cluster, an increase in gene expression in response to n-hexadecane was found for CYP153 (15-fold induction; p < 0.05). Although not statistically significant, the ferredoxin gene showed 2.3-fold induction. Transcripts for the ferredoxin reductase gene were not detected in our RT-qPCR assays.

## Discussion

The assumption that composting could be a source of microorganisms with capacity to degrade hydrocarbons was based on previous observations that composting harbors a great diversity of biodegraders carrying metabolic pathways to degrade a plethora of complex organic compounds [16,17,21–27,54,55]. With this rationale we succeeded in the isolation of two *Gordonia* strains from compost that have the ability to degrade n-hexadecane. *Gordonia* spp. are known for their broad capability to degrade recalcitrant organic compounds, including hydrocarbons [10,11].

The isolation of fastidious microorganisms from environmental samples represents a challenge. The chosen strategy to recover distinct microorganisms was based on the use of two methods of sample preparation, with or without a previous decontamination step, allied to the selective culture in presence of n-hexadecane, which is similar to the methodology used to isolate other hexadecane degrading-bacteria [56]. All *Gordonia* isolates were obtained from the decontamination method due to the cell wall characteristics that make these microorganisms resistant to treatment with chemical agents, demonstrating the success of the chosen strategy. The exclusive recovery of bacteria of the *Gordonia* genus when we used the decontamination method possibly reflects its effectiveness in eliminating other Gram positive and Gram negative fast- and slow-growing bacteria.

The qualitative analysis of n-hexadecane degradation by DCPIP test was chosen as a screening test. Nine isolates were evaluated for the ability to degrade n-hexadecane by the 2,6-DCPIP qualitative test. These isolates were chosen because, to our knowledge, these species have not been reported in human or animal infections, a desirable feature for a potential use as a remediation microorganism. The six *Gordonia* isolates showed color change of 2,6-DCPIP solution, demonstrating abilities to degrade n-hexadecane. A strain of *G*. *paraffinivorans* (HD321) isolated from an oil-producing well in China was shown to have the ability to grow in paraffin, an aliphatic hydrocarbon [57]. Moreover *G*. *sihwensis* strain S9, which was isolated from raw sludge in a wastewater treatment facility in the USA [58], has been reported as a possible hydrocarbon-degrader based on genomic analysis. To date, none of these species have experimental data demonstrating the degradation of n-hexadecane, a major constituent of gasoline and important polluting agent due to spills at gas stations [45]. Thus, the isolates MTZ052 and MTZ096 identified respectively as *G*. *paraffinivorans* and *G*. *sihwensis* were chosen for quantitative analysis of their hydrocarbon degradation ability.

The CG-MS tests performed in this work with the isolates of *G*. *paraffinivorans* (MTZ052) and *G*. *sihwensis* (MTZ096) showed distinct n-hexadecane degradation rates in 28 days, 86% and 100% respectively, and confirm the 2,6-DCPIP assay as a useful primary screening test as reported [33].

The genome sequences showed that the MTZ052 isolate encodes only the CYP153 system while the MTZ96 harbors both the AH and the CYP153 systems, suggesting that the n-hexadecane biodegradation process is more efficient when the CYP and AH systems work together. Also, our data suggest that the CYP153 and AH systems effectively play a role in alkane degradation, since the transcription of both gene clusters increased in the presence of the substrate, in both species. Lo Piccolo and collaborators performed experiments to quantify the expression of AH system genes of *Gordonia* sp. SoCg and reported that the expression of *alkB*, *rubA*3, *rubA*4 and *rubB* genes were induced in the presence of n-hexadecane, with *alkU* as the only gene in the system that presented no significant change [59]. Whether these systems are related to degradation efficiency and conversion of these carbon sources remains to be determined. In another study, Liang et al. (2016) characterized CYP153 expression pattern in *Dietzia* sp. by cloning a LacZ reporter gene downstream of the CYP153 promoter and verified that indeed, expression is induced by alkane in the substrate as carbon source [60]. The lack of expression of ferredoxin reductase in the CYP153 cluster in MTZ052 suggests that this gene is expressed at very low levels or that another ferredoxin gene, outside of the cluster, may have this function.

To the best of our knowledge, this is the first report demonstrating the ability of *G*. *paraffinivorans* MTZ052 and *G*. *sihwensis* MTZ096 to degrade n-hexadecane. Subsequent studies are necessary to evaluate the ability of these strains to remediate water and soil environments contaminated by hydrocarbon pollutants.

## Supporting information

S1 Table Primers used in the qPCR assay. (DOC)

S2 Table Digital DNA-DNA hybridization (dDDH) and Mummer-implemented Average Nucleotide Identity (ANIm) values for the *Gordonia* MTZ052 and MTZ096 isolates. (DOCX)

S3 Table List of 57 homologues identified by the software Get_homolog among *Gordonia sp.*. (DOC)

S1 Fig GC–MS total ion chromatogram showing n-hexadecane biodegradation by the MTZ052 e MTZ096 isolates and negative control of degradation. Time of analysis: 0h; 72h; 14, 21 and 28 days.
(TIF)
