# Supplementary material for: Characterization of novel hydrocarbon-degrading Gordonia paraffinivorans and Gordonia sihwensis strains isolated from composting
Source: PLoS One. 2019 Apr 18;14(4):e0215396. doi: 10.1371/journal.pone.0215396 (PMC6472744; doi:10.1371/journal.pone.0215396)
Supplement: S1 Table — (DOC) [file pone.0215396.s001.doc]

**Supplemental material**

**Table S1: Primers used in the qPCR assay**

| Primer | Gene | Sequence (5' - 3') | Sense | Concentration | Amplicon |
| --- | --- | --- | --- | --- | --- |
| PRCP060 | Ferredoxin MTZ052 | CGTTCGTCGAGGGACAGAAC | F | 4 µm | 187 |
| PRCP061 |  | TGGCAATCGGGATTCATCTC | R | 4 µm |  |
| PRCP062 | CYP153 MTZ052 | GACCTCATCAGCATGTTG | F | 800 ηM | 321 |
| PRCP063 |  | GTACCACATCACGAGCTTG | R | 800 ηM |  |
| PRCP064 | Ferredoxin redutase MTZ052 | ACTTCTATCGCCGCATCCAC | F | 2 µm | 235 |
| PRCP065 |  | GCATCGACGACGATGCCGTC | R | 2 µm |  |
| PRCP078 | Ferredoxin redutase MTZ096 | GATCATCTTGGACTTGCCGC | F | 600 ηM | 328pb |
| PRCP079 |  | CGCTGGTCGAAGCTCTATC | R | 600 ηM |  |
| PRCP080 | CYP153 MTZ096 | GAGGCGTACCACATCACTAC | F | 600 ηM | 333pb |
| PRCP081 |  | CGGCTTCGATCTGATCACC | R | 600 ηM |  |
| PRCP082 | Ferredoxin MTZ096 | GTTCGACGATCAAGCCGTCC | F | 600 ηM | 228pb |
| PRCP083 |  | TGCAGGTCGCGACCAACAAC | R | 600 ηM |  |
| PRCP084 | *Alk*B MTZ096 | CGTCTCCAAGATCGGTCTG | F | 600 ηM | 343pb |
| PRCP085 |  | CTTCAGGACGTCGTTGCTCG | R | 300 ηM |  |
| PRCP086 | *rub*A3 MTZ096 | CGAAGTGTGCGAGTACGTC | F | 600 ηM | 134pb |
| PRCP087 |  | CGAAATCGATCTTCTCGCGG | R | 600 ηM |  |
| PRCP088 | *rub*A4 MTZ096 | CTTCCGTTGCATGCAGTGC | F | 600 ηM | 151pb |
| PRCP089 |  | CTCGACCATCTCGAAGTCG | R | 600 ηM |  |
| PRCP092 | *Alk*U MTZ096 | GCAGGCAGACCGTGTACAAC | F | 600 ηM | 317pb |
| PRCP093 |  | GTCGATGGCGATCCACGAC | R | 600 ηM |  |
| PRCP096 | 16S | GTATTACCGCGGCTGCTGGC | F | 600 ηM | 183 pb |
| PRCP097 |  | CCAGACTCCTACGGGAGGCAGC | R | 300 ηM |  |
| PRCP098 | *Rub* MTZ096 | GCTGTCGAAGGATCTGCTC | F | 600 ηM | 333pb |
| PRCP099 |  | AGTTCCAGGCCGATCAGCC | R | 600 ηM |  |
