# Supplementary material for: Characterization of novel hydrocarbon-degrading Gordonia paraffinivorans and Gordonia sihwensis strains isolated from composting
Source: PLoS One. 2019 Apr 18;14(4):e0215396. doi: 10.1371/journal.pone.0215396 (PMC6472744; doi:10.1371/journal.pone.0215396)
Supplement: S2 Table — (DOCX) [file pone.0215396.s002.docx]

**Supplemental Material**

**Table S2:** **Digital DNA-DNA hybridization (dDDH) and Mummer-implemented Average Nucleotide Identity (ANIm) values for the *Gordonia* MTZ052 and MTZ096 isolates**

| **dDDH** | | | | | |
| --- | --- | --- | --- | --- | --- |
|  | *G. paraffinivorans* NBRC 108238 | ***G. paraffinivorans* MTZ052** | *G. sihwensis* NBRC 108236 | *G. sihwensis* no. 9 | ***G. sihwensis* MTZ096** |
| *G. paraffinivorans* NBRC 108238 | 100 | 85.90 | 21.00 | 21.60 | 21.10 |
| ***G. paraffinivorans* MTZ052** | 85.90 | 100 | 21.20 | 21.70 | 21.10 |
| *G. sihwensis* NBRC 108236 | 21.00 | 21.20 | 100 | 92.50 | 95.10 |
| *G. sihwensis* no. 9 | 21.60 | 21.70 | 92.50 | 100 | 92.10 |
| ***G. sihwensis* MTZ096** | 21.10 | 21.10 | 95.10 | 92.10 | 100 |
|  |  |  |  |  |  |
| **ANIm** | | | | | |
|  | *G. paraffinivorans* NBRC 108238 | ***G. paraffinivorans* MTZ052** | *G. sihwensis* NBRC 108236 | *G. sihwensis* no. 9 | ***G. sihwensis* MTZ096** |
| *G. paraffinivorans* NBRC 108238 | 1.0 | 0.9857480459848078 | 0.8470219982281402 | 0.8512970067864005 | 0.8462879970390873 |
| ***G. paraffinivorans* MTZ052** | 0.9857480459848078 | 1.0 | 0.8479231769700976 | 0.853765466236651 | 0.8478114831925362 |
| *G. sihwensis* NBRC 108236 | 0.8470219982281402 | 0.8479231769700976 | 1.0 | 0.9900025278565783 | 0.9930355774623922 |
| *G. sihwensis* no. 9 | 0.8512970067864005 | 0.853765466236651 | 0.9900025278565783 | 1.0 | 0.987020347634759 |
| ***G. sihwensis* MTZ096** | 0.8462879970390873 | 0.8478114831925362 | 0.9930355774623922 | 0.987020347634759 | 1.0 |
