# Supplementary material for: Characterization of novel hydrocarbon-degrading Gordonia paraffinivorans and Gordonia sihwensis strains isolated from composting
Source: PLoS One. 2019 Apr 18;14(4):e0215396. doi: 10.1371/journal.pone.0215396 (PMC6472744; doi:10.1371/journal.pone.0215396)
Supplement: S3 Table — (DOC) [file pone.0215396.s003.doc]

**Table S3: List of 57 homologues identified by the software Get_homolog among *Gordonia sp***

| 1. 30S ribosomal protein S14 type Z - rpsZ 2. 30S ribosomal protein S15 - rpsO 3. 30S ribosomal protein S17 - rpsQ 4. 30S ribosomal protein S1 - rpsA 5. 30S ribosomal protein S20 - rpsT 6. 30S ribosomal protein S7 - rpsG 7. 30S ribosomal protein S8 - rpsH 8. 3-isopropylmalate dehydratase small subunit - leuD 9. 50S ribosomal protein L14 - rplN 10. 50S ribosomal protein L22 - rplV 11. 50S ribosomal protein L27 - rpmA 12. 50S ribosomal protein L29 - rpmC 13. 50S ribosomal protein L35 - rpmI 14. 7Fe ferredoxin 15. Acetolactate synthase small subunit - ilvN 16. Acyl-CoA desaturase 17. Adenosylhomocysteinase - ahcY 18. ATP-dependent Clp protease adapter protein ClpS 19. ATP synthase subunit alpha 20. Chromosome partitioning protein ParA 21. Elongation factor LepA 22. Elongation factor P - efp 23. Elongation factor Tu - tuf 24. Glucosyl-3-phosphoglycerate synthase - gpgS 25. Glyceraldehyde-3-phosphate dehydrogenase - gap 26. Hypoxanthine phosphoribosyltransferase - hpt 27. Imidazoleglycerol-phosphate dehydratase - hisB 28. Iron-sulfur cluster assembly protein SufB 29. Nitrogen regulatory protein P-II - glnK2 30. O-succinylhomoserine sulfhydrylase - metZ 31. Peptidyl-prolyl cis-trans isomerase 32. Putative ABC transporter ATP-binding protein 33. Putative GTP-binding protein 34. Putative HIT family protein 35. Putative iron-sulfur cluster assembly accessory protein 36. Putative methyltransferase 37. Putative TetR family transcriptional regulator 38. Putative thiosulfate sulfurtransferase 39. Putative two-component response regulator 40. Seryl-tRNA synthetase - serS 41. Signal recognition particle protein - ffh 42. Single-stranded DNA-binding protein - ssb 43. Succinate dehydrogenase cytochrome b subunit - sdhC 44. Succinate dehydrogenase iron-sulfur protein - sdhB 45. Thioredoxin reductase - trxB 46. Two-component response regulator RegX3 47. Tyrosyl-tRNA synthetase - tyrS 48. Hypothetical protein 49. Hypothetical protein 50. Hypothetical protein 51. Hypothetical protein 52. Hypothetical protein 53. Hypothetical protein 54. Hypothetical protein 55. Hypothetical protein 56. Hypothetical protein 57. Hypothetical protein |
| --- |
