# Supplementary figures and images for: Characterization of novel hydrocarbon-degrading Gordonia paraffinivorans and Gordonia sihwensis strains isolated from composting
Source: PLoS One. 2019 Apr 18;14(4):e0215396. doi: 10.1371/journal.pone.0215396 (PMC6472744; doi:10.1371/journal.pone.0215396)

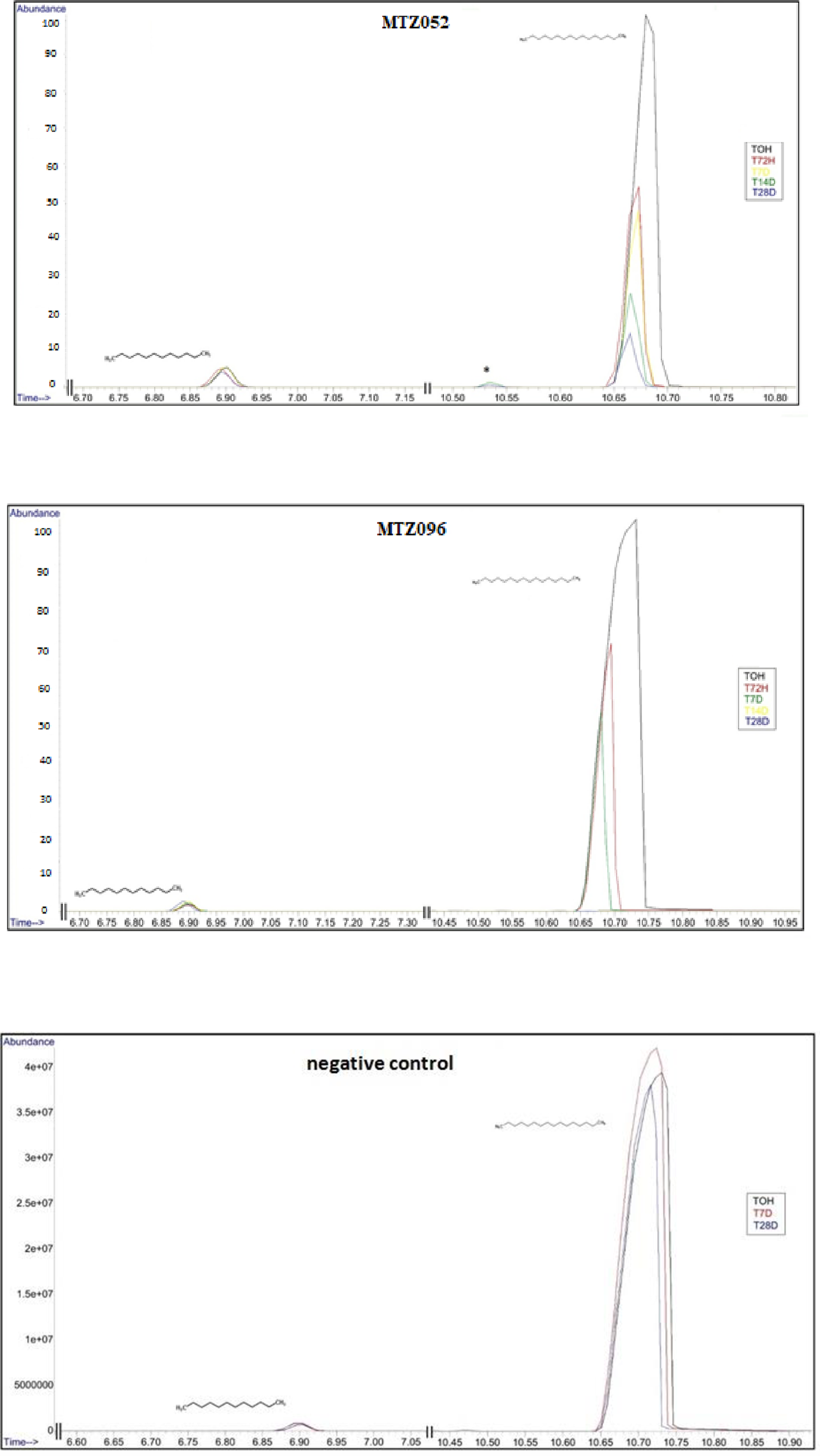

Supplement: S1 Fig — Time of analysis: 0h; 72h; 14, 21 and 28 days. (TIF) [file pone.0215396.s004.tif]
